# The Probing Behavior Component of Disease Transmission in Insect-Transmitted Bacterial Plant Pathogens

**DOI:** 10.3390/insects10070212

**Published:** 2019-07-19

**Authors:** Timothy A. Ebert

**Affiliations:** Department of Entomology and Nematology, Citrus Research and Education Center, University of Florida, IFAS, 700 Experiment Station Road, Lake Alfred, FL 33850, USA; tebert@ufl.edu

**Keywords:** EPG, electropenetrography, electrical penetration graph, citrus

## Abstract

Insects can be effective vectors of plant diseases and this may result in billions of dollars in lost agricultural productivity. New, emerging or introduced diseases will continue to cause extensive damage in afflicted areas. Understanding how the vector acquires the pathogen and inoculates new hosts is critical in developing effective management strategies. Management may be an insecticide applied to kill the vector or a host plant resistance mechanism to make the host plant less suitable for the vector. In either case, the tactic must act before the insect performs the key behavior(s) resulting in either acquisition or transmission. This requires knowledge of the timing of behaviors the insect uses to probe the plant and commence ingestion. These behaviors are visualized using electropenetrography (EPG), wherein the plant and insect become part of an electrical circuit. With the tools to define specific steps in the probing process, we can understand the timing of acquisition and inoculation. With that understanding comes the potential for more relevant testing of management strategies, through insecticides or host plant resistance. The primary example will be *Candidatus* Liberibacter asiaticus transmitted by *Diaphorina citri* Kuwayama in the citrus agroecosystem, with additional examples used as appropriate.

## 1. Introduction

About 150 out of 7100 described bacterial species are phytopathogens [1]. These diseases are most problematic in tropical and subtropical regions where environmental conditions favor bacterial growth. Crop losses can be extensive. *Candidatus* Liberibacter asiaticus (*C*Las), the putative causal agent of Huanglongbing, was first detected in Florida 2005 [2], and the disease spread rapidly through the citrus growing regions of the state. Starting from the 1997–1998 season, the average orange yield through the 2004–2005 season was 897 boxes per hectare. Production was 462 boxes per hectare for the 2016–2017 season, or 48% less than the pre-*C*Las average. Grapefruit production has suffered similarly, with a pre-*C*Las average of 998 boxes per hectare and a 2016–2017 season production of 568 boxes per hectare (43% reduction) (www.nass.usda.gov/Statistics_by_State/Florida/Publications/Citrus/Citrus_Statistics). The effect of reduced yields is amplified by a loss of planted acreage: 73% loss in orange and 39% loss in grapefruit. By one estimate, the economic consequences for all citrus production in Florida (orange, grapefruit, tangerine, and others) between the 2012–2013 season and the 2015–2016 season were such that Florida has lost 4.4 billion (US Dollars) and 7945 full-time or seasonal jobs in this three-year span [3].

In a 2012 survey, the top ten plant bacterial pathogens were identified along with three runners-up via a survey of authors who have published in the journal *Molecular Plant Pathology*. There were two pathogens from this list where an insect acts as the primary vector: *Candidatus* Liberibacter asiaticus (rank 13) and *Xylella fastidiosa* (rank 8) [4]. The former is transmitted by the psyllid *Diaphorina citri* Kuwayama (Hemiptera: Liviidae), and possibly by *Trioza erytreae* (Del Guercio) (Hemiptera: Triozidae) [5]. In addition to these psyllids, there are six other species of *Diaphorina*, and five other psyllid species that attack citrus [6]. None of these have been ruled out as possible vectors of *C*Las. In contrast, *X. fastidiosa* is transmitted by several sharpshooters (Hemiptera: Cicadellidae), some spittlebugs (Hemiptera: Cercopidae) and cicadas (Hemiptera: Cicadidae). In the case of *C*Las, the primary means to manage the disease is through vector control along with destroying infected trees, limiting movement by establishing quarantine zones, and providing disease-free nursery stock [7]. This strategy is typical of most vector borne bacterial plant pathogens.

Initially, the insect vector is easily managed using off-the-shelf insecticides that have been used to manage other insect pests. However, this tool provides at best short-term solutions to the problem because the vectors develop resistance. Furthermore, the pesticides are tested for efficacy primarily through topical or residue bioassays that are focused on lethality: e.g., *D. citri* [8]. If the time it takes for a pesticide to kill the vector is long enough, then the vector can still salivate into the plant and transmit the disease [9]. Thus, the insecticide reduces the transmission rate to some extent [10], or may not work at all [11], despite being lethal in the long run. Bioassays that go beyond simple dose-mortality are needed to better align insecticidal activity to disease management through insect vector control. In vector management for the control of a phloem limited bacteria, the key question is whether the insecticide stops salivation into the phloem. There is only one tool that is ideally suited to determine if salivation into the phloem has occurred.

## 2. Electropenetrography

**Figure 1:**
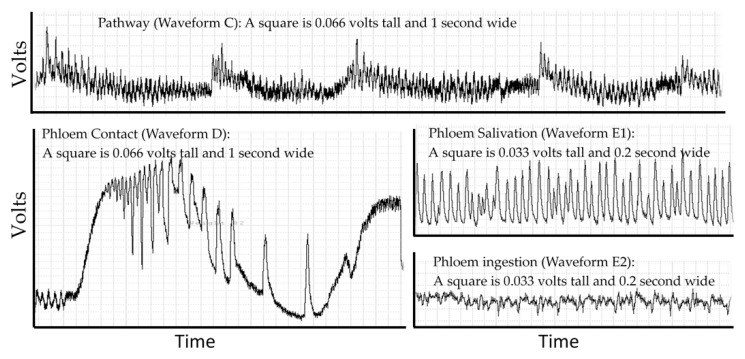
The repetitive patterns for four waveforms in the *D. citri*. Pathway waveform (C), where stylets are moving through leaf cuticle. Phloem contact (waveform D) has large changes in voltage, creating a stereotypical set of large spikes that gradually diminish in frequency and can then transition into phloem salivation (waveform E1). E1 can transition into phloem ingestion (waveform E2). The displayed waveforms occurred in a span of 200 seconds within a probe that lasted 4 h.

Probing behavior is monitored using electropenetrography (or an electrical penetration graph, EPG). This technique is the only real-time non-lethal approach to monitoring such behavior in opaque plant or animal tissues that obscures the location of the insect stylets. EPG overcomes this problem by making the host and insect a part of an electrical circuit via an electropenetrograph [12,13]. The circuit is complete only if some part of the insect (legs, ovipositor, or mouthparts) contacts the host. The resulting signal is generated by changes in resistance and by physical and biochemical processes that generate electrical potentials (abbreviated EMF) [14]. Resistance changes as the insect salivates onto or into the host, and electrons are then able to pass through the salivary canal. Resistance also changes depending on the type of saliva. Typically, the saliva of sheath feeding hemipterans (like *D. citri*) is classified as gelling saliva or watery saliva [15], but this simple classification hides a diverse biochemical composition derived from both the insect and endosymbionts (aphids [16]; *D. citri* [17,18]). Electrons also pass through the food canal when the insect ingests host fluids. Electricity is generated as fluids move in the narrow tubes of the stylets and is generated through muscle contractions in the insect [14,19]. There may also be signals from the part of the plant damaged by the herbivore to other parts of the plant [20]. The EPG recording is a combined signal from all sources showing diagnostic repetitive patterns from these processes that can be correlated to specific behaviors using histology, transparent artificial diets, transmission studies and other techniques. Electropenetrographs can be either alternating current (AC) or direct current (DC) depending on whether the electricity applied to the insect is AC or DC [21], and a new model can switch between AC or DC [22]. The result, regardless of the machine used for measurement, is a recording that is a sequence of repetitive patterns (Figure 1). Different insect species will have different patterns and allocate their time to each behavior in different proportions.

EPG offers a unique real-time view of the interactions taking place at the junction of the plant, the vector, and the pathogen, that is useful for understanding the biological interactions and provides opportunities for direct assessment of biological consequences when the system is manipulated. While the primary use of EPG to date is in studying the probing behavior of hemipterans (aphids, psyllids, whiteflies, sharpshooters, etc.), it has been used elsewhere; grasshoppers [23]; mites [24]; thrips [25,26]; ticks [27]; and mosquitoes [28]. It has also been used to assess signaling in plants as they are wounded mechanically or by herbivores [29]. There are also possibilities for exploring how entomopathogens or parasitoids influence aspects of a pathosystem at the point of parasitization, or as the natural enemy develops within the host. Because the bacterial pathosystems are complex networks of several organisms interacting at different levels, it is useful to focus on one system as an example of how EPG is a critical tool in understanding these systems. The example pathosystem discussed herein will be the phloem-limited Gram-negative circulative propagative alpha-proteobacteria *Candidatus* Liberibacter asiaticus (*C*Las) transmitted by the Asian citrus psyllid, *D. citri* to *Citrus* and other rutaceous hosts. While other reviews of this pathosystem exist [7,30,31,32], the importance of the probing behavior of the insect is missing from these discussions.

## 3. *Candidatus* Liberibacter asiaticus

The *C*Las pathosystem is a collection of linked lifecycles of the psyllid vector, the bacterial plant pathogen, the host plant, and various bacterial, fungal, and viral organisms that are typically associated with the insect [33] or plant. The system is too complex to study in one research project, yet the cost of studying very small aspects of the system is that the links are lost. For the vector side of this pathosystem, most of the interactions must pass through the stylets of the vector, because that is the only way that plant fluids are transferred from the plant to the insect. EPG is the only non-destructive approach for observing the behavioral events taking place in this process. Any event that changes the health of the vector or plant—or that changes the ability of the vector to assimilate ingested nutriment—should have an effect on probing behavior. Initially, the effect will be compensatory feeding to restore nutritional homeostasis [34], but eventually conditions may become so extreme that compensatory feeding is insufficient to prevent reductions in survivorship and fecundity. While some of the interactions may have an effect that is so minor as to be indistinguishable from background variability in probing behavior, other interactions will not be so cryptic. However, there is no EPG data within any pathosystem that clearly ranks all of the possible interactions and their effect on vector biology. This is especially problematic because, even in well studied systems, new species are still being identified and not all individuals within a psyllid population have all of the possible species [35,36]. Despite such difficulties, current knowledge is leveraged to develop the best understanding possible of the multitrophic interactions typical of hemipteran vectors. In the following details, it is assumed that no detail is unimportant if it affects the exposure of the vector to *C*Las or influences the health of the vector for good or ill.

### 3.1. CLas Adults

**Figure 2:**
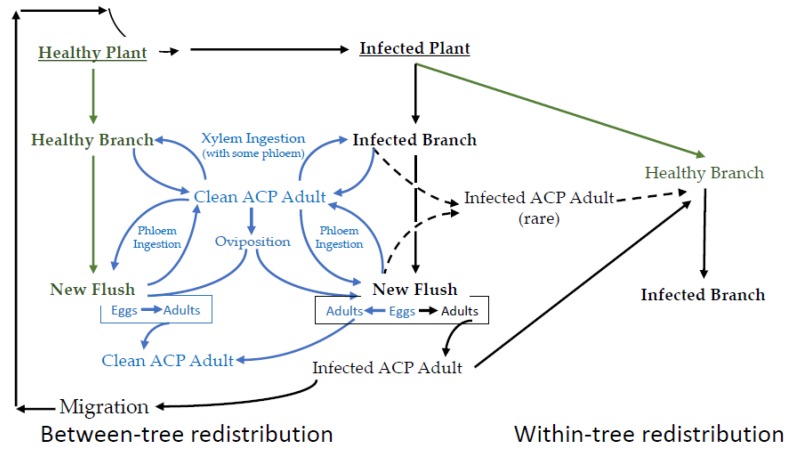
A simplified representation of the sequence of major events in the disease cycle for the *C*Las-*D. citri*-citrus pathosystem. Not all branches on an infected tree are infected. Not all psyllids (adult or nymph) ingesting phloem on an infected branch become infected. Not all infected psyllids can inoculate. Healthy psyllids are in blue. Healthy plants are ingreen. Infected plants and psyllids are in black.

EPG data are best understood in the context of the interconnected lifecycles of the vector, endosymbionts, pathogen, and the plant host. A simplified schematic representation of the epidemiology of *C*Las transmission is provided in Figure 2, wherein the integrated and cyclic nature of these processes is emphasized. In a *C*Las-free citrus growing region, psyllids complete their life cycle without becoming infected. This part of Figure 2 has little bearing on disease transmission, except that it increases the pool of potential vectors. The first infected citrus tree occurs after either the migration of an infected adult or the importation of diseased plants into an area where the vector is already established. While it is possible for a healthy adult to feed on an infected plant and thereby acquire *C*Las, nymphs that acquire *C*Las have from 10 to 100 times the bacterial load 15 days post-eclosion, compared to psyllids that acquired the bacteria as adults [37]. The most consistent route for acquisition is by late instars. Immature psyllids that acquire CLas are more capable of transmitting relative to psyllids that acquire *C*Las as adults [37,38,39,40], and adults that acquired *C*Las as late instars transmit *C*Las 43.4% of the time, relative to psyllids that acquire *C*Las as adults that transmit 11.8% [41].

Adult *D. citri* can be infected with *C*Las by acquiring it at any of several stages in the insect’s life cycle. As adults, about 4% of females can acquire *C*Las when mating with *C*Las-infected males [40].

### 3.2. CLas Eggs

Female *D. citri* lay eggs exclusively at the apical meristem of new growth [42,43]. This is the only location where 1st instar *D. citri* can survive. About 3.6% of progeny are infected with *C*Las through transovarial transmission [39].

### 3.3. CLas Nymphs

The 1st instar nymphs are found close to the apical meristem. Later instars can move short distances and are able to survive on more mature parts of the plant [44]. The 1st through 3rd instars cannot acquire *C*Las, or do so rarely (it was not observed to occur, but the 95% confidence interval was from 0% to 10%), while 4th and 5th instars have acquisition rates of 11% and 20% respectively (95% confidence interval of from 3% to 27% and 8% to 40% respectively) [45] (Data were reanalyzed using package binGroupin R). There are several reasons why young nymphs may not acquire *C*Las. Early instars are restricted to the growing meristem. The bacterial load within a few micrometers of the growing point may not be sufficient to reliably infect the early instars. It is expected that the total volume of fluid ingested by early instars is less than for later instars or adults [46]. If bacteria are present at low concentrations and the early instar ingests a lesser volume before molting, then there is less opportunity for acquisition. The early instars molt into the next instar after a few days and this does not give *C*Las enough time within one instar to replicate in the vector to reliably detectable levels. While PCR methods can detect a single target-DNA sequence, the methodology for purifying the DNA, resuspending it, and taking a sample from this suspension, makes it unlikely that a single copy of the target DNA will be detected. Finally, Ammar pointed out that the diameters of the food canal and salivary canal may be insufficient for transmission in early instars. Acquisition is through the food canal that is 460 ± 37.6 nm in diameter for the 1st instar *D. citri* compared to the adult at 872.5 ± 62.5 nm [47]. *C*Las is pleomorphic with rod and spherical forms. The rod is from 2600 to 6300 nm long and between 330 and 660 nm in diameter, while the spherical form is between 860 and 1500 nm in diameter [48]. For inoculation, *C*Las must pass through the salivary canal and that is 212 ± 17.9 nm in the 1st instar versus 379.5 ± 34.7 in adults [47]. We have not found published measurements of *C*Las in salivary glands, but the size of the bacteria in vector ovaries was from 390 to 670 nm long with a diameter of between 190 and 390 nm for rods and 610 and 800 nm for spheres [40]. Even if the 1st through 3rd instars can acquire at some low rates, their role in within-tree spread of *C*Las is unimportant, because the early instars are effectively sedentary as they are unable to survive on more mature parts of the plant.

#### 3.3.1. Changes in Behavior

Later instar nymphs are better able to acquire CLas with acquisition rates of up to 100% being reported [39]. This could be due to a larger diameter food canal, a longer duration of an instar that would provide more time for CLas to replicate before the next molt, or to changes in probing behavior. The 5th instar *D. citri* have an average of 9 bouts of phloem ingestion in 42 h with an average phloem ingestion time of 5.58 h per bout. In contrast, adults have 5 bouts lasting an average of 1.42 h [49]. Changes in behavior between adults and nymphs have been shown in other systems, though nymphs do not always have longer ingestion periods [50,51]. However, in the CLas system, late instar nymphs have greater exposure to CLas due to longer periods of phloem ingestion [49].

#### 3.3.2. Changes in Physiology

CLas and endosymbionts replicate in the psyllid. This takes resources, and all of these resources must pass through the food canal in the stylets. The physiological changes may include physical damage to the insect, and it is likely that some of these changes modify psyllid probing behavior. Following ingestion, *C*Las colonizes the midgut epithelial cells of *D. citri* causing cell death and the generation of endoplasmic reticulum associated bodies that *C*Las then colonizes [52,53]. However, the degree of cellular damage to the midgut is less in the 5th instar *D. citri* than in adults [54]. After penetrating the midgut, a biofilm is formed on the midgut membrane [30]. *Wolbachia* is an alpha-bacterium that is commonly found in insects, and it serves in a parasitic or mutualistic capacity and can be found in the psyllid midgut. Despite the changes in *D. citri* midgut cells caused by *C*Las, there were no upregulated or downregulated *Wolbachia* proteins detected as a result of *C*Las infection [55]. However, a different study found that 26 *Wolbachia* proteins were downregulated in CLas-infected psyllids [56], and many more changes have been reported [57]. It is possible that *Wolbachia* helps *C*Las by secreting a protein that inhibits the phage lytic cycle in *C*Las [58], but there was no difference in *Wolbachia* copy number in two isofemale lines that differed significantly in ability to transmit *C*Las (0% versus 24% transmission) [59]. While *Wolbachia* is a well-studied bacterium in insects, there are at least 12 other bacterial species that can be recovered from the psyllid gut and the abundance of these bacteria is highly variable [36]. Wherever found, all symbionts must extract the energy and nutrients they need to reproduce from the host insect or nutriment that the insect has ingested. Furthermore, if the host experiences cell damage, then additional resources will be needed to repair the damage and in the case of damage to the gut, there may be a reduced ability to extract nutriment from the plant sap. Two studies have reported EPG results consistent with a “hungrier psyllid” hypothesis associated with *C*Las infection [60,61]. After acquisition, *C*Las replicates in the midgut epithelial cells, enters the hemolymph, and thence moves to the salivary glands. Inoculation occurs when the psyllid salivates into the phloem. However, completing these steps is not guaranteed and therefore the ability to acquire *C*Las is no guarantee that the psyllid will be equally successful at transmitting *C*Las [59].

There are several possible reasons why nymphs that acquire CLas become more efficient vectors. Among the reasons are that the immune system is less developed in nymphs relative to adults, as shown by a greater metabolic response in adults to *C*Las infection [55]. Furthermore, the endosymbiont community changes throughout development. *Diaphorina citri* has three endosymbionts: *Candidatus* Carsonella rudii, *Candidatus* Profftella armature, and *Wolbachia pipientis*. The presence of *C*Las was correlated with reduced populations of *Candidatus* Carsonella rudii and *Wolbachia pipientis*. *Candidatus* Profftella armature levels were reduced in females but increased in males [62]. However, another study found no difference in *Wolbachia* populations between CLas-infected versus healthy psyllids [56] or *Wolbachia* populations increased [63]. In general, populations of endosymbionts increase as the psyllid matures [64,65] with the highest populations found in adults [66]. However, some data indicate that the relationship is more a function of time rather than specifically related to life stage of *D. citri* [66]. The relative abundance of these endosymbionts is *Profftella* > *Carsonella* > *Wolbachia* [66]. *Candidatus* Carsonella rudii play a nutritional role in *D. citri* [67]), while *Candidatus* Profftella armature is involved in defense [68] and both are mostly in a bacteriocyte in the psyllid abdomen [64]. In contrast, *Wolbachia* is mostly located in the midgut [54]. The number of proteins in the hemolymph from these endosymbionts was always reduced when *C*Las was also present [57]. However, two isofemale lines that differed in their ability to acquire and transmit had no difference in endosymbiont abundance [59].

### 3.4. Plant Physiology

There are physiological changes in an infected host plant that influence the psyllid’s ability to maintain a balanced diet and the insect can respond through a process termed compensatory feeding [69,70] to maintain an optimal nutriment intake. *C*Las alters the levels of many proteins, amino acids, organic acids, sugars, and other constituents of the infected host plant, and the response changes depending on cultivar [71,72,73], though plants both infected with *C*Las and infected with the psyllid may moderate such responses [73]. Healthy psyllid adults reduced probing from 74% of probe time spent in phloem-related activities (D + E1 + E2, see Figure 1) on healthy plants, to 8% on heavily infected plants [74]. A different group reported little effect on feeding behavior due to the infection status of the host plant, but found a significant difference in how nymphs responded versus adults [49]. *C*Las infection causes many changes in phloem composition and these changes should have some effect on the psyllid’s probing behavior. For comparison, there are considerable differences in phloem composition between citrus cultivars and such differences result in changes in probing behavior that may have fitness costs [75]. Differences in phloem composition are also correlated with suitability of the plant to support *C*Las [76]. Thus, one might ask either why cultivar differences in phloem composition result in changes in psyllid probing behavior while the changes in phloem composition from *C*Las infection do not always have an effect, or what features of the experimental methodology can result in a failure to detect the significant changes in probing behavior.

### 3.5. Experimental Design Issues

Just because a tree tests positive for *C*Las does not mean that all branches of the tree are infected (Figure 2) [77], and even a positive PCR test is no guarantee that viable bacteria are present [78]. There is some probability that a healthy psyllid can lay eggs on an infected tree and still have healthy progeny. Even if the psyllid progeny come from an infected branch, there is still no guarantee that all of the progeny will be infected. On the insect side, a PCR+ outcome does not guarantee that the psyllid has either acquired or is able to transmit. It is possible that there are a few target DNA fragments in the gut of the psyllid (giving a PCR+ outcome). Further, not all individuals have the same vector competency and some psyllids are good vectors (acquisition rate in the range 28–32%, inoculation rate in the range 19–28%) while others are poor vectors (acquisition rate in the range 5–8%, inoculation rate in the range 0–3%) [59]. Furthermore, there was no clear relationship between a high probability of acquiring *C*Las and a high probability of being able to transmit *C*Las. Given these issues, EPG techniques can assist research by enabling a correction for the amount of time spent salivating or ingesting phloem as part of experiments that would otherwise involve exposing a host plant to a vector(s) for a fixed time interval.

### 3.6. EPG as a Tool

While it may seem obvious that inoculation takes place at phloem salivation and acquisition at phloem ingestion for a phloem-limited pathogen, this can be proven by using an EPG monitor. The monitor is used to determine when to remove an insect from a healthy host plant based on the performance of a specific behavior, as shown by the EPG monitor. The EPG recording can show that the insect did not perform other behaviors during periods when the scientist was away from the equipment. Healthy *D. citri* adults exposed to *C*Las-infected plants were unable to acquire or transmit if feeding was interrupted before contact with the phloem [79]. No acquisition of *C*Las was detected, as long as insects did not perform phloem ingestion (assessed from 50 individual insects that have only pathway (waveform C), 50 insects that have pathway and phloem contact (waveforms C and D), 50 insects with C+D and phloem salivation (C + D + E1)). Three of 50 insects with at least 1 h of phloem ingestion tested positive for *C*Las [79]. This outcome is consistent with the idea that *C*Las is phloem limited.

Wu et al. (2016) tested inoculation by allowing 62 *C*Las-infected psyllids to probe while monitoring them using EPG. Only 14 plants out of 62 were infected, and on all but one of these 14 plants, the psyllid performed phloem salivation. The performance of other behaviors did not increase the transmission rate. In the lone exception, the psyllid performed only pathway and xylem ingestion behaviors, but did not access phloem tissues [61].

EPG can be used to identify walking versus resting/probing behavior in *D. citri* [80] because of strong tarsal contact with the plant. This approach was used to improve understanding of how kaolin clay (sold as Surround WP) works as a pest management tool. The kaolin coating makes the plant slippery and the clay particles cover the tarsal claws, thereby preventing the claws from holding the plant. The result is that the insect has difficulty with inserting its mouthparts into the plant [81,82].

In developing resistant cultivars, it is important to understand how the vector interacts with the host plant, because changes in probing behavior will affect transmission rates. Even if both cultivars are susceptible, the difference may be important to developing more precise models of disease spread through different citrus growing regions. Psyllids were placed on either Valencia or Midsweet sweet orange, *Citrus sinensis* [83]. While both Valencia and Midsweet can serve as effective hosts for rearing this insect, there were significant differences in the feeding behavior. Psyllids on Valencia took longer to reach xylem ingestion than psyllids on Midsweet, and psyllids on Valencia spent less time ingesting from the phloem. Psyllids on Midsweet spent more time in phloem contact (waveform D), and in phloem salivation (waveform E1). However, there was no significant difference in psyllids on these hosts in the time it took to reach phloem ingestion (waveform E2), the number of E2 events, or the duration of phloem ingestion. It would seem that they get the same level of nutrition by changing the balance of other behaviors to overcome the physical or chemical differences between Valencia and Midsweet sweet orange. The important points here are that there are cultivar-related differences in the probing behavior that are relevant to the acquisition and transmission of *C*Las, and EPG methodology is sensitive enough to detect cultivar differences. EPG will therefore be an effective tool in rapid testing of host plant resistance or tolerance in a plant breeding program. Furthermore, since the critical tissue is new growth, EPG could be used to test tissue-cultured plants for host plant resistance or tolerance.

### 3.7. Relative Importance of Feeding Site

Adult psyllids in the field can be found on all available parts of the plant (leaves, flowers, stems, fruit), but that does not equate to equal vector competence. When population levels are low, most psyllids are on the underside (abaxial surface) of leaves. When population levels are high, the psyllids are mostly on the abaxial surface of leaves, but some can be found on the upper (adaxial) leaf surface. Psyllids are frequently on new growth, but adults can be found on mature leaves when psyllid populations are high or during seasons when new growth is absent. While there was a significant effect of adaxial versus abaxial leaf surface, it was minor compared to the difference in the behavior between immature and mature leaves. *Diaphorina citri* primarily ingests from the phloem (mean duration of phloem ingestion 4.3 h: mean duration of xylem ingestion 0.5 h in a 24 h recording) on immature leaves and primarily ingestion from the xylem (mean duration of phloem ingestion 0.4 h: mean duration of xylem ingestion 1.5 h) on mature leaves [84]. A difference between immature and mature leaves can also be observed in phloem salivation frequency and duration, with psyllids on immature leaves performing an average 3.8 salivation events per insect and a mean duration of 1.7 min, while psyllids on mature leaves performed only 1.4 salivation events with a mean duration of 0.7 min per event. Thus, experiments on transmission where the plant lacks new flush have shown reduced acquisition and transmission rates [85,86].

### 3.8. Psyllid Nutrition

The psyllid is clearly not spending all its time ingesting from the phloem or the xylem. In one experiment [84], a great deal of time was spent sitting on the plant (between 40% and 50% of total time) or probing (27% to 38%), and only 19% to 27% of the time was spent ingesting phloem sap. Another experiment contrasted the nymphs, which spend 65% to 79% of their time ingesting phloem and 5% to 12% of their time on non-probing activities versus the adults that spend 14% to 24% of their time ingesting phloem and 43% to 44% of their time on non-probing activities [49]. If growth and reproduction are nutrient limited, then the most efficient solution would be to find the phloem and remain there for longer periods of time. Given that the adults have time to ingest more food but do not take advantage of this time, it is unlikely that nutrition alone is limiting the population growth rate of the psyllid. Rather*, D. citri* ingestion duration and frequency are balanced against several competing forces: nutriment acquisition, osmotic regulation, and the processing of plant defensive chemistries against the metabolic requirements to maintain physiological homeostasis, reproduce, and find new hosts. If ingestion occurs too rapidly, toxins accumulate faster than they are metabolized, and the insect dies. The insect can reduce the rate of ingestion of the nutrient-rich phloem to match detoxification rates, or it can ingest the xylem to dilute toxins or adjust osmotic balance. To simply maintain life, the psyllid’s primary issue is water loss, and the psyllid deals with this by ingesting xylem with brief periods of phloem ingestion for physiological maintenance. With new growth and expected reproduction, additional resources are needed, and the psyllid ingests more phloem, but still short of a maximum acquisition rate. Consistent with this, psyllids on mature leaves spend more time ingesting the xylem (90% xylem, 10% phloem), while psyllids on immature leaves spend more time ingesting the phloem (81% phloem, 19% xylem) [84]. Population growth rates are limited by the amount of phloem the psyllid can ingest—given that the psyllid will also ingest more toxins—and the rate at which food can be assimilated and thence converted to eggs.

### 3.9. Cell Count

In the AC-DC monitor, there is the option of setting an internal resistor to values ranging from 10^6^ to 10^13^ ohms. The choice influences the proportion of the recorded signal that is due to changes in resistance versus changes in EMF [22]. At 10^6^, the recorded signal is mostly influenced by changes in resistance, while at 10^13^, the recorded signal is mostly EMF. Changing the setting of the internal resistor is used to help understand the biological source of the waveforms [22]. In the *C*Las system, there was an expectation that careful examination of the EPG output would enable one to correlate a waveform for *D. citri* ingesting a *C*Las cell. This event has not yet been discernable at an input resistance of 10^9^, but, recording at other input resistances might be more informative. The ability to detect such events assumes that the bacterial cell will have a higher resistance than an electrolyte solution thereby changing resistance, or that *C*Las would alter the rate of flow through the food canal thereby changing EMF. The latter makes some sense because the size of the bacterium is at least 1/3 the diameter of the food canal and 87% of the diameter of the salivary canal. The ability to count the number of cells acquired or transmitted would be useful in determining the number of *C*Las necessary to overcome host defenses. This would improve understanding of vector competence in individual *D. citri* and enable a more detailed study of defensive capability in the host plant.

## 4. Other Bacterial Pathosystems

The relationships between host plant, pathogen, and vector involve specific molecular interactions for recognition, defense, and offense. In all cases, there are specific probing behaviors associated with ingestion where the vector acquires the pathogen, while other behaviors are associated with inoculation. However, different insects generate different waveforms and researchers use different naming conventions to describe these waveforms. Thus, waveform C (pathway) in *D. citri* is not the same as waveform C (xylem ingestions) in sharpshooters. For this reason, descriptions of behaviors are used rather than waveform names when referencing the probing behavior of different species.

### 4.1. Xylella fastidiosa

The Gram-negative xylem-limited non-circulative propagative gamma-proteobacterium *Xylella fastidiosa* was ranked as the more serious of the two insect-transmitted bacterial pathogens [4]. This system was reviewed recently [12,87,88,89,90]. Reproduction in the vector takes place on the cuticular surface of the anterior foregut [91]. While nymphs can acquire *X. fastidiosa*, the bacteria are lost at each molt as the cuticle of the foregut is shed. Acquisition can be achieved either by trial ingestion or sustained ingestion [12]. While longer ingestion events improve acquisition rates, this has not been quantified. Ingested *X. fastidiosa* cells attach first to the cibarium and in heavy infections, these bacteria grow into the pre-cibarium and other areas [91]. The primary pathway for inoculation occurs by discharge egestion where the pre-cibarium is colonized and the insect clears this area by egesting a mix of plant cell contents and saliva into xylem tissues. This behavior is nearly exclusively done in xylem [12]. Sharpshooters can act as “flying syringes” and inoculation takes place when several *Xyllela* cells are briefly held in the food canal of the stylets and then egested with saliva in a new probe [92]. However, with many thousands of insects per hectare, even a rare occurrence will happen occasionally. As a non-selective mode of transmission, it is likely that this pathway will be found in many other pathosystems. Thus, if a technique like RNAi were used to prevent *C*Las from recognizing the psyllid midgut epithelium, the psyllid might still transmit *C*Las as a flying syringe, albeit at greatly reduced efficiency.

### 4.2. Candidatus Phytoplasma

Phytoplasma diseases are a diverse group of phloem-limited bacterial pathogens transmitted by insects in the families Cicadellidae (leafhopper), Cixiidae, Delphacidae, Derbidae (planthoppers), and Psyllidae (psyllids) [93]. As with *C*Las, adults that acquire as nymphs are more effective vectors. Once acquired, the phytoplasma passes through the gut wall into the hemolymph and thence to the salivary glands [94]. Unlike with *C*Las, there are some clear differences in transmission among the sexes. Aster yellows is a phytoplasma disease transmitted by *Macrosteles quadrilineatus* Forbes (Hemiptera: Cicadellidae). Males can be up to twice as likely to acquire the pathogen, but 55% of the infected females could inoculate while only 35% of infected males inoculate individual lettuce leaves. In a 4-plant arena, females infect 18% of the plants compared to an 8% infection rate for males, and the difference grows to 30% for females and 10% for males when the spatial scale was increased to an entire greenhouse containing many pants. Males move more frequently and therefore there is less opportunity to transmit [95]. In contrast, *Scaphoideus titanus* Ball males were better vectors of *Candidatus* Phytoplasma vitis than were females at least, in part, because males spent more time in the phloem compared to females. Furthermore, females spent more time in the xylem than the phloem, while males spent their time evenly in the phloem and the xylem [96,97].

## 5. Environmental Change

The problems with plant pathogens are changing as CO_2_ levels and temperatures increase. The nutritional quality of the plants will change, as will the relative proportion of defensive compounds in plants under elevated CO_2_ [98,99]. Metabolic rates will change under elevated temperatures and the balance of endosymbionts may also change [100,101,102,103]. These factors will all impact vector and pathogen biology, as each component of the system struggles to find a new level of homeostasis. Probing behaviors will change and therefore so will the rate at which the vector acquires and inoculates the plant pathogen (virus: [104]). These factors have the potential to rearrange the agroecosystem landscape and necessitate redesigning best management practices to address changing circumstances.

## 6. Conclusions

Electropenetrography provides a unique view into the biology of many insect pests and helps improve understanding of how these insects further their impact through the transmission of plant pathogens. No other technique provides this information in real time without damaging the insect or plant. EPG can be used either to understand the biology of the insect in a multitrophic system that includes a host (plant or animal), and pathogens of the insect or host whether they are bacterial, viral, fungal, or something else. While EPG informs on only one part of insect behavior, it is the critical part that is otherwise not observable, yet is the bottleneck through which must pass most of the nutriment necessary for survival. While EPG can be used to better understand the biology of these systems, it can also be a monitoring tool used in support of other techniques. Bioassays examining mortality as an endpoint can now include how quickly the insecticide stops probing and thereby prevent transmission. Bioassays that look at infection by exposing healthy host plants to a vector for a fixed time interval can adjust individual outcomes for the proportion of exposure time spent on the behavior wherein the vector inoculates the host plant. Imprecision in such bioassays increases the variability in outcome, thereby making it more difficult to interpret the data or detect treatment differences. Using EPG as a monitoring tool overcomes this issue, no matter if the “treatment” involves a search for different cultivars, more effective insecticides, or assessing the efficacy of newer approaches like RNAi.

Probing behavior is the critical juncture between the phytophagous hemipteran insect vector and its host plant. Changes in the biological system can have a direct influence on feeding behavior through behavioral manipulation, or an indirect effect through changes in nutritional balance leading to a hungrier psyllid. In turn, these changes influence migration probabilities by altering perceptions of host suitability, and the availability of resources for reproduction. Altered reproductive rates as well as altered migration behavior influences the spread of pathogens through the geographic range of the host plant. Furthermore, the individual variability in vector competence acting through interlinked lifecycles of the bacterial pathogen, its vector, and the host plant, result in additional variability in the spread of the pathogen.

The host plant and the vector each have defenses that limit the ability of a bacterial pathogen to colonize and reproduce. For this reason, it is unlikely, though possible, for a single bacterial cell to overcome all the host defenses, replicate, and be available for transfer between the vector and plant. Thus, increasing titer in the plant improves the probability of acquisition by the vector, and increasing titers in the relevant parts of the vector improves the probability of inoculation resulting in disease. As life cycles become more complex, the biochemical interactions also become more complex, because there are more steps in the disease cycle and more opportunities to defend against such attack. All these steps result in biochemical changes that influence the nutritional needs of the vector, the nutritional quality of the plant for the vector, and thereby the probing behavior of the vector. In turn, this alters the ability of the vector to acquire, reproduce, migrate, and transmit the disease. The complex interactions between the vector, plant host, and pathogen, provide many unique opportunities for biotechnology to influence the system. However, the complex lifecycles often have minor alternate paths, like the flying syringe that could become more common given the right selection pressures. There may even be events so rare that they are undetectable by experiments using only a few hundred individuals. It is therefore likely that biotechnology solutions will ultimately have the same problems with the development of resistance as we have found with all chemical pesticides (antibiotics, insecticides, herbicides, fungicides). The advantage of biotechnology is that it offers unique modes of action, is highly selective, and can target key steps that may not be directly lethal. In this capacity, biotechnology could provide a key element in integrated pest management programs, but key to understanding how these systems work is to understand how insect probing behavior influences the within-plant and between-plant distribution of the pathogen.
